# Supplementary material for: Modeling glioblastoma heterogeneity as a dynamic network of cell states
Source: Mol Syst Biol. 2021 Sep 16;17(9):e10105. doi: 10.15252/msb.202010105 (PMC8444284; doi:10.15252/msb.202010105)
Supplement: Supplementary file 6 — Source Data for Figure 5 [file MSB-17-e10105-s004.zip › Figure5A_sourcedata/GSEA_3071/nosigdb_stateI.GseaPreranked.1622795145664/DOROSZKO_U3180MG_CLUSTER_3.html]

Details for gene set DOROSZKO\_U3180MG\_CLUSTER\_3[GSEA]

|  || Dataset | state13071 |
| Phenotype | NoPhenotypeAvailable |
| Upregulated in class | na\_neg |
| GeneSet | DOROSZKO\_U3180MG\_CLUSTER\_3 |
| Enrichment Score (ES) | -0.5691199 |
| Normalized Enrichment Score (NES) | -3.56287 |
| Nominal p-value | 0.0 |
| FDR q-value | 0.0 |
| FWER p-Value | 0.0 |
Table: GSEA Results Summary

  

Fig 1: Enrichment plot: DOROSZKO\_U3180MG\_CLUSTER\_3      
 Profile of the Running ES Score & Positions of GeneSet Members on the Rank Ordered List

  

| PROBE | GENE SYMBOL | GENE\_TITLE | RANK IN GENE LIST | RANK METRIC SCORE | RUNNING ES | CORE ENRICHMENT || 1 | SLC20A1 |  |  | 116 | 1.035 | -0.0315 | No |
| 2 | PHGDH |  |  | 279 | 0.699 | -0.0792 | No |
| 3 | PSMB9 |  |  | 437 | 0.560 | -0.1261 | No |
| 4 | TALDO1 |  |  | 455 | 0.545 | -0.1287 | No |
| 5 | KDELR2 |  |  | 737 | 0.401 | -0.2156 | No |
| 6 | ARPC1B |  |  | 743 | 0.400 | -0.2152 | No |
| 7 | P4HB |  |  | 748 | 0.398 | -0.2145 | No |
| 8 | SHMT2 |  |  | 777 | 0.390 | -0.2214 | No |
| 9 | FUCA2 |  |  | 797 | 0.383 | -0.2255 | No |
| 10 | PSMA3 |  |  | 866 | 0.361 | -0.2452 | No |
| 11 | LY6E |  |  | 1030 | 0.321 | -0.2951 | No |
| 12 | JPT2 |  |  | 1037 | 0.321 | -0.2954 | No |
| 13 | DEGS1 |  |  | 1178 | 0.293 | -0.3382 | No |
| 14 | IDH2 |  |  | 1324 | 0.264 | -0.3827 | No |
| 15 | TCEAL9 |  |  | 1348 | 0.260 | -0.3887 | No |
| 16 | CCDC34 |  |  | 1376 | 0.255 | -0.3960 | No |
| 17 | NDUFA6 |  |  | 1420 | -0.250 | -0.4083 | No |
| 18 | MSH2 |  |  | 1438 | -0.252 | -0.4124 | No |
| 19 | CEP152 |  |  | 1446 | -0.253 | -0.4134 | No |
| 20 | RALY |  |  | 1448 | -0.253 | -0.4124 | No |
| 21 | CHAC2 |  |  | 1449 | -0.253 | -0.4112 | No |
| 22 | RAD1 |  |  | 1475 | -0.255 | -0.4178 | No |
| 23 | CCND3 |  |  | 1482 | -0.255 | -0.4184 | No |
| 24 | VOPP1 |  |  | 1490 | -0.256 | -0.4194 | No |
| 25 | NUP85 |  |  | 1492 | -0.256 | -0.4184 | No |
| 26 | MMD |  |  | 1511 | -0.258 | -0.4228 | No |
| 27 | NR2C2AP |  |  | 1527 | -0.260 | -0.4263 | No |
| 28 | MRPL37 |  |  | 1535 | -0.261 | -0.4272 | No |
| 29 | CTPS1 |  |  | 1540 | -0.261 | -0.4271 | No |
| 30 | RBBP4 |  |  | 1549 | -0.262 | -0.4284 | No |
| 31 | CHAF1B |  |  | 1554 | -0.262 | -0.4283 | No |
| 32 | TMEM54 |  |  | 1555 | -0.262 | -0.4270 | No |
| 33 | ARL6IP6 |  |  | 1577 | -0.265 | -0.4323 | No |
| 34 | SUPT16H |  |  | 1588 | -0.266 | -0.4342 | No |
| 35 | HNRNPF |  |  | 1636 | -0.272 | -0.4477 | No |
| 36 | SAE1 |  |  | 1638 | -0.273 | -0.4466 | No |
| 37 | SMC3 |  |  | 1650 | -0.274 | -0.4487 | No |
| 38 | SNX5 |  |  | 1663 | -0.275 | -0.4512 | No |
| 39 | BUB3 |  |  | 1687 | -0.278 | -0.4570 | No |
| 40 | TEDC1 |  |  | 1698 | -0.279 | -0.4588 | No |
| 41 | SCARA3 |  |  | 1701 | -0.280 | -0.4581 | No |
| 42 | RAD54L |  |  | 1702 | -0.280 | -0.4567 | No |
| 43 | RPA1 |  |  | 1717 | -0.282 | -0.4597 | No |
| 44 | PEG10 |  |  | 1734 | -0.284 | -0.4633 | No |
| 45 | KIF2C |  |  | 1735 | -0.284 | -0.4619 | No |
| 46 | SAAL1 |  |  | 1736 | -0.284 | -0.4605 | No |
| 47 | PSMC3 |  |  | 1752 | -0.287 | -0.4638 | No |
| 48 | MIS18BP1 |  |  | 1764 | -0.289 | -0.4658 | No |
| 49 | CENPJ |  |  | 1768 | -0.290 | -0.4653 | No |
| 50 | CASP8AP2 |  |  | 1857 | -0.300 | -0.4917 | No |
| 51 | CENPQ |  |  | 1860 | -0.300 | -0.4908 | No |
| 52 | FAF1 |  |  | 1927 | -0.309 | -0.5101 | No |
| 53 | KPNA2 |  |  | 1944 | -0.312 | -0.5136 | No |
| 54 | SRR |  |  | 1950 | -0.312 | -0.5137 | No |
| 55 | SPATA33 |  |  | 1972 | -0.314 | -0.5187 | No |
| 56 | GTF3A |  |  | 1977 | -0.315 | -0.5184 | No |
| 57 | SUZ12 |  |  | 2011 | -0.318 | -0.5273 | No |
| 58 | PARPBP |  |  | 2026 | -0.322 | -0.5301 | No |
| 59 | DDX39A |  |  | 2043 | -0.324 | -0.5335 | No |
| 60 | ACYP1 |  |  | 2052 | -0.326 | -0.5344 | No |
| 61 | TRAP1 |  |  | 2055 | -0.326 | -0.5334 | No |
| 62 | SNRPB |  |  | 2064 | -0.328 | -0.5343 | No |
| 63 | MZT2B |  |  | 2075 | -0.330 | -0.5359 | No |
| 64 | CCDC15 |  |  | 2080 | -0.331 | -0.5355 | No |
| 65 | AHCY |  |  | 2089 | -0.333 | -0.5363 | No |
| 66 | ARPC5L |  |  | 2094 | -0.333 | -0.5359 | No |
| 67 | CHEK1 |  |  | 2108 | -0.336 | -0.5384 | No |
| 68 | ZNF367 |  |  | 2131 | -0.339 | -0.5436 | No |
| 69 | MRM2 |  |  | 2145 | -0.342 | -0.5461 | No |
| 70 | CCT2 |  |  | 2163 | -0.345 | -0.5497 | No |
| 71 | COPS3 |  |  | 2175 | -0.348 | -0.5514 | No |
| 72 | MZT2A |  |  | 2183 | -0.349 | -0.5519 | No |
| 73 | YEATS4 |  |  | 2187 | -0.349 | -0.5511 | No |
| 74 | POC1A |  |  | 2199 | -0.351 | -0.5529 | No |
| 75 | MXD3 |  |  | 2201 | -0.352 | -0.5514 | No |
| 76 | PKMYT1 |  |  | 2214 | -0.356 | -0.5534 | No |
| 77 | ENOPH1 |  |  | 2217 | -0.357 | -0.5523 | No |
| 78 | ARRB2 |  |  | 2235 | -0.360 | -0.5559 | No |
| 79 | SMC1A |  |  | 2240 | -0.361 | -0.5553 | No |
| 80 | PAFAH1B3 |  |  | 2264 | -0.365 | -0.5608 | No |
| 81 | BCL7C |  |  | 2274 | -0.366 | -0.5618 | No |
| 82 | MTHFD1 |  |  | 2282 | -0.369 | -0.5622 | No |
| 83 | DEPDC1B |  |  | 2288 | -0.370 | -0.5619 | No |
| 84 | TUBB4B |  |  | 2291 | -0.370 | -0.5607 | No |
| 85 | GLO1 |  |  | 2292 | -0.371 | -0.5589 | No |
| 86 | TIMM10 |  |  | 2296 | -0.372 | -0.5580 | No |
| 87 | YWHAQ |  |  | 2304 | -0.373 | -0.5583 | No |
| 88 | DONSON |  |  | 2316 | -0.375 | -0.5599 | No |
| 89 | CKS1B |  |  | 2332 | -0.380 | -0.5628 | No |
| 90 | BUB1B |  |  | 2335 | -0.381 | -0.5615 | No |
| 91 | MCM2 |  |  | 2349 | -0.383 | -0.5637 | No |
| 92 | CKAP2 |  |  | 2357 | -0.385 | -0.5640 | No |
| 93 | ZWILCH |  |  | 2358 | -0.385 | -0.5621 | No |
| 94 | MSH6 |  |  | 2362 | -0.386 | -0.5611 | No |
| 95 | PSIP1 |  |  | 2364 | -0.386 | -0.5595 | No |
| 96 | MEA1 |  |  | 2386 | -0.390 | -0.5642 | No |
| 97 | CSNK2B |  |  | 2391 | -0.390 | -0.5635 | No |
| 98 | RDX |  |  | 2399 | -0.392 | -0.5638 | No |
| 99 | CENPW |  |  | 2402 | -0.393 | -0.5624 | No |
| 100 | UHRF1 |  |  | 2404 | -0.393 | -0.5608 | No |
| 101 | PRIM2 |  |  | 2409 | -0.394 | -0.5601 | No |
| 102 | WDR34 |  |  | 2414 | -0.395 | -0.5594 | No |
| 103 | HAUS1 |  |  | 2423 | -0.397 | -0.5600 | No |
| 104 | H2AFV |  |  | 2431 | -0.398 | -0.5602 | No |
| 105 | HPF1 |  |  | 2435 | -0.399 | -0.5591 | No |
| 106 | NCAPH2 |  |  | 2443 | -0.400 | -0.5594 | No |
| 107 | POLA2 |  |  | 2452 | -0.401 | -0.5599 | No |
| 108 | CKS2 |  |  | 2466 | -0.404 | -0.5620 | No |
| 109 | PDCD5 |  |  | 2476 | -0.406 | -0.5628 | No |
| 110 | HAUS8 |  |  | 2494 | -0.412 | -0.5661 | No |
| 111 | ECI1 |  |  | 2500 | -0.414 | -0.5656 | No |
| 112 | CSE1L |  |  | 2512 | -0.417 | -0.5670 | Yes |
| 113 | PGRMC1 |  |  | 2513 | -0.417 | -0.5650 | Yes |
| 114 | UBA2 |  |  | 2517 | -0.418 | -0.5638 | Yes |
| 115 | NME4 |  |  | 2518 | -0.418 | -0.5617 | Yes |
| 116 | RACGAP1 |  |  | 2522 | -0.419 | -0.5606 | Yes |
| 117 | NFATC2IP |  |  | 2531 | -0.423 | -0.5610 | Yes |
| 118 | CACYBP |  |  | 2536 | -0.424 | -0.5602 | Yes |
| 119 | BAZ1B |  |  | 2538 | -0.425 | -0.5584 | Yes |
| 120 | PARP2 |  |  | 2542 | -0.425 | -0.5572 | Yes |
| 121 | SLF1 |  |  | 2551 | -0.427 | -0.5576 | Yes |
| 122 | TEX30 |  |  | 2556 | -0.428 | -0.5567 | Yes |
| 123 | POLE3 |  |  | 2570 | -0.432 | -0.5587 | Yes |
| 124 | H2AFZ |  |  | 2572 | -0.433 | -0.5569 | Yes |
| 125 | LRR1 |  |  | 2579 | -0.434 | -0.5566 | Yes |
| 126 | SSRP1 |  |  | 2580 | -0.434 | -0.5544 | Yes |
| 127 | TMEM106C |  |  | 2592 | -0.437 | -0.5557 | Yes |
| 128 | ANP32B |  |  | 2593 | -0.438 | -0.5535 | Yes |
| 129 | GGCT |  |  | 2610 | -0.442 | -0.5564 | Yes |
| 130 | UBR7 |  |  | 2611 | -0.442 | -0.5542 | Yes |
| 131 | NCAPG2 |  |  | 2621 | -0.445 | -0.5548 | Yes |
| 132 | PPIF |  |  | 2624 | -0.446 | -0.5532 | Yes |
| 133 | HNRNPA3 |  |  | 2634 | -0.449 | -0.5538 | Yes |
| 134 | HIRIP3 |  |  | 2635 | -0.449 | -0.5516 | Yes |
| 135 | DCK |  |  | 2636 | -0.449 | -0.5494 | Yes |
| 136 | DUT |  |  | 2640 | -0.450 | -0.5481 | Yes |
| 137 | DCTPP1 |  |  | 2644 | -0.451 | -0.5468 | Yes |
| 138 | MASTL |  |  | 2646 | -0.452 | -0.5448 | Yes |
| 139 | EXOSC8 |  |  | 2659 | -0.456 | -0.5463 | Yes |
| 140 | PAQR4 |  |  | 2663 | -0.460 | -0.5450 | Yes |
| 141 | CCNA2 |  |  | 2667 | -0.462 | -0.5436 | Yes |
| 142 | NME1 |  |  | 2669 | -0.463 | -0.5417 | Yes |
| 143 | MND1 |  |  | 2682 | -0.468 | -0.5431 | Yes |
| 144 | KIF22 |  |  | 2683 | -0.468 | -0.5408 | Yes |
| 145 | CMC2 |  |  | 2694 | -0.470 | -0.5416 | Yes |
| 146 | MMS22L |  |  | 2698 | -0.471 | -0.5402 | Yes |
| 147 | MCM6 |  |  | 2699 | -0.471 | -0.5379 | Yes |
| 148 | SNRPD1 |  |  | 2701 | -0.471 | -0.5358 | Yes |
| 149 | HAT1 |  |  | 2703 | -0.472 | -0.5338 | Yes |
| 150 | PRC1 |  |  | 2705 | -0.472 | -0.5317 | Yes |
| 151 | FANCD2 |  |  | 2707 | -0.474 | -0.5297 | Yes |
| 152 | CENPN |  |  | 2708 | -0.474 | -0.5273 | Yes |
| 153 | MELK |  |  | 2709 | -0.474 | -0.5250 | Yes |
| 154 | SLC43A3 |  |  | 2713 | -0.475 | -0.5236 | Yes |
| 155 | TUBG1 |  |  | 2715 | -0.475 | -0.5215 | Yes |
| 156 | NDC80 |  |  | 2723 | -0.478 | -0.5213 | Yes |
| 157 | EXO1 |  |  | 2728 | -0.479 | -0.5202 | Yes |
| 158 | RPA3 |  |  | 2730 | -0.480 | -0.5181 | Yes |
| 159 | SHCBP1 |  |  | 2733 | -0.480 | -0.5164 | Yes |
| 160 | PIGX |  |  | 2736 | -0.482 | -0.5146 | Yes |
| 161 | BRIP1 |  |  | 2739 | -0.482 | -0.5128 | Yes |
| 162 | CHCHD3 |  |  | 2749 | -0.485 | -0.5133 | Yes |
| 163 | TIMELESS |  |  | 2752 | -0.487 | -0.5115 | Yes |
| 164 | UNG |  |  | 2758 | -0.489 | -0.5106 | Yes |
| 165 | CENPF |  |  | 2761 | -0.490 | -0.5088 | Yes |
| 166 | ACTL6A |  |  | 2762 | -0.492 | -0.5063 | Yes |
| 167 | BRCA2 |  |  | 2772 | -0.495 | -0.5067 | Yes |
| 168 | NUF2 |  |  | 2781 | -0.497 | -0.5068 | Yes |
| 169 | SKA1 |  |  | 2787 | -0.498 | -0.5059 | Yes |
| 170 | SLBP |  |  | 2792 | -0.499 | -0.5046 | Yes |
| 171 | MGME1 |  |  | 2797 | -0.500 | -0.5034 | Yes |
| 172 | DTYMK |  |  | 2798 | -0.501 | -0.5009 | Yes |
| 173 | WDR76 |  |  | 2800 | -0.501 | -0.4987 | Yes |
| 174 | RAD18 |  |  | 2802 | -0.501 | -0.4966 | Yes |
| 175 | RBL1 |  |  | 2808 | -0.503 | -0.4956 | Yes |
| 176 | HNRNPAB |  |  | 2816 | -0.508 | -0.4953 | Yes |
| 177 | SIVA1 |  |  | 2822 | -0.510 | -0.4943 | Yes |
| 178 | ASF1B |  |  | 2824 | -0.510 | -0.4921 | Yes |
| 179 | DSCC1 |  |  | 2828 | -0.512 | -0.4905 | Yes |
| 180 | CDCA3 |  |  | 2829 | -0.513 | -0.4880 | Yes |
| 181 | TMEM97 |  |  | 2834 | -0.514 | -0.4867 | Yes |
| 182 | SPC24 |  |  | 2836 | -0.514 | -0.4844 | Yes |
| 183 | HMGB3 |  |  | 2839 | -0.516 | -0.4825 | Yes |
| 184 | TOP2A |  |  | 2842 | -0.517 | -0.4805 | Yes |
| 185 | E2F8 |  |  | 2847 | -0.519 | -0.4792 | Yes |
| 186 | LIG1 |  |  | 2848 | -0.520 | -0.4766 | Yes |
| 187 | WDHD1 |  |  | 2853 | -0.522 | -0.4753 | Yes |
| 188 | NABP2 |  |  | 2854 | -0.522 | -0.4727 | Yes |
| 189 | CKAP2L |  |  | 2857 | -0.523 | -0.4707 | Yes |
| 190 | NUDT1 |  |  | 2859 | -0.524 | -0.4684 | Yes |
| 191 | RFC5 |  |  | 2862 | -0.526 | -0.4664 | Yes |
| 192 | CDK2 |  |  | 2863 | -0.526 | -0.4638 | Yes |
| 193 | TRIP13 |  |  | 2865 | -0.526 | -0.4615 | Yes |
| 194 | RAD21 |  |  | 2866 | -0.527 | -0.4589 | Yes |
| 195 | HJURP |  |  | 2869 | -0.527 | -0.4569 | Yes |
| 196 | ANP32E |  |  | 2877 | -0.530 | -0.4564 | Yes |
| 197 | POLR2I |  |  | 2887 | -0.534 | -0.4566 | Yes |
| 198 | FAM111A |  |  | 2917 | -0.552 | -0.4631 | Yes |
| 199 | ARHGAP11A |  |  | 2921 | -0.556 | -0.4612 | Yes |
| 200 | CMSS1 |  |  | 2931 | -0.561 | -0.4613 | Yes |
| 201 | ATAD5 |  |  | 2935 | -0.563 | -0.4594 | Yes |
| 202 | PIN1 |  |  | 2936 | -0.565 | -0.4566 | Yes |
| 203 | FANCA |  |  | 2937 | -0.565 | -0.4538 | Yes |
| 204 | H2AFY |  |  | 2942 | -0.568 | -0.4522 | Yes |
| 205 | CENPH |  |  | 2945 | -0.569 | -0.4500 | Yes |
| 206 | OLA1 |  |  | 2946 | -0.569 | -0.4472 | Yes |
| 207 | MIS18A |  |  | 2947 | -0.570 | -0.4443 | Yes |
| 208 | DSN1 |  |  | 2949 | -0.571 | -0.4418 | Yes |
| 209 | MAD2L2 |  |  | 2952 | -0.573 | -0.4396 | Yes |
| 210 | KIF4A |  |  | 2953 | -0.574 | -0.4367 | Yes |
| 211 | TACC3 |  |  | 2956 | -0.575 | -0.4345 | Yes |
| 212 | CENPX |  |  | 2958 | -0.576 | -0.4319 | Yes |
| 213 | KIF23 |  |  | 2961 | -0.579 | -0.4297 | Yes |
| 214 | C3orf14 |  |  | 2962 | -0.580 | -0.4268 | Yes |
| 215 | H1FX |  |  | 2968 | -0.581 | -0.4255 | Yes |
| 216 | PIMREG |  |  | 2971 | -0.583 | -0.4232 | Yes |
| 217 | NCAPH |  |  | 2974 | -0.585 | -0.4209 | Yes |
| 218 | GGH |  |  | 2975 | -0.585 | -0.4180 | Yes |
| 219 | CTNNAL1 |  |  | 2976 | -0.585 | -0.4151 | Yes |
| 220 | POP7 |  |  | 2980 | -0.588 | -0.4131 | Yes |
| 221 | OLFM1 |  |  | 2981 | -0.589 | -0.4102 | Yes |
| 222 | VRK1 |  |  | 2983 | -0.592 | -0.4075 | Yes |
| 223 | SMC2 |  |  | 2984 | -0.592 | -0.4046 | Yes |
| 224 | SKA3 |  |  | 2985 | -0.592 | -0.4016 | Yes |
| 225 | KNL1 |  |  | 2986 | -0.592 | -0.3987 | Yes |
| 226 | PAXX |  |  | 2999 | -0.602 | -0.3995 | Yes |
| 227 | TPX2 |  |  | 3006 | -0.606 | -0.3983 | Yes |
| 228 | C19orf48 |  |  | 3013 | -0.608 | -0.3972 | Yes |
| 229 | SNRNP25 |  |  | 3018 | -0.613 | -0.3954 | Yes |
| 230 | TCF19 |  |  | 3019 | -0.613 | -0.3924 | Yes |
| 231 | RTKN2 |  |  | 3020 | -0.613 | -0.3893 | Yes |
| 232 | GINS4 |  |  | 3021 | -0.615 | -0.3862 | Yes |
| 233 | METRN |  |  | 3024 | -0.615 | -0.3838 | Yes |
| 234 | TFDP1 |  |  | 3027 | -0.617 | -0.3814 | Yes |
| 235 | DNMT1 |  |  | 3035 | -0.622 | -0.3805 | Yes |
| 236 | LSM3 |  |  | 3037 | -0.625 | -0.3777 | Yes |
| 237 | PARP1 |  |  | 3039 | -0.626 | -0.3749 | Yes |
| 238 | LRRCC1 |  |  | 3047 | -0.631 | -0.3739 | Yes |
| 239 | RBBP8 |  |  | 3053 | -0.637 | -0.3723 | Yes |
| 240 | AURKB |  |  | 3056 | -0.639 | -0.3698 | Yes |
| 241 | PHF19 |  |  | 3060 | -0.643 | -0.3675 | Yes |
| 242 | LMNB2 |  |  | 3061 | -0.644 | -0.3643 | Yes |
| 243 | NCAPD3 |  |  | 3064 | -0.646 | -0.3617 | Yes |
| 244 | ALYREF |  |  | 3068 | -0.649 | -0.3595 | Yes |
| 245 | SAC3D1 |  |  | 3073 | -0.655 | -0.3575 | Yes |
| 246 | FANCI |  |  | 3074 | -0.656 | -0.3542 | Yes |
| 247 | C21orf58 |  |  | 3078 | -0.659 | -0.3518 | Yes |
| 248 | CDC45 |  |  | 3081 | -0.661 | -0.3492 | Yes |
| 249 | GTSE1 |  |  | 3083 | -0.663 | -0.3462 | Yes |
| 250 | CDCA4 |  |  | 3084 | -0.663 | -0.3429 | Yes |
| 251 | TMPO |  |  | 3088 | -0.666 | -0.3405 | Yes |
| 252 | MAD2L1 |  |  | 3099 | -0.675 | -0.3403 | Yes |
| 253 | RFC3 |  |  | 3108 | -0.683 | -0.3394 | Yes |
| 254 | KIFC1 |  |  | 3110 | -0.684 | -0.3363 | Yes |
| 255 | LSM4 |  |  | 3115 | -0.688 | -0.3342 | Yes |
| 256 | PA2G4 |  |  | 3116 | -0.690 | -0.3307 | Yes |
| 257 | DBF4 |  |  | 3117 | -0.690 | -0.3273 | Yes |
| 258 | MYBL1 |  |  | 3119 | -0.691 | -0.3242 | Yes |
| 259 | LMNB1 |  |  | 3121 | -0.692 | -0.3210 | Yes |
| 260 | POLD3 |  |  | 3122 | -0.695 | -0.3176 | Yes |
| 261 | SMC4 |  |  | 3126 | -0.699 | -0.3150 | Yes |
| 262 | CENPK |  |  | 3127 | -0.700 | -0.3116 | Yes |
| 263 | RFC4 |  |  | 3129 | -0.701 | -0.3084 | Yes |
| 264 | MCM5 |  |  | 3132 | -0.706 | -0.3055 | Yes |
| 265 | NFIC |  |  | 3133 | -0.707 | -0.3020 | Yes |
| 266 | GINS1 |  |  | 3137 | -0.713 | -0.2994 | Yes |
| 267 | GMNN |  |  | 3140 | -0.719 | -0.2964 | Yes |
| 268 | GAMT |  |  | 3141 | -0.723 | -0.2928 | Yes |
| 269 | KIF15 |  |  | 3142 | -0.723 | -0.2892 | Yes |
| 270 | BTG3 |  |  | 3148 | -0.727 | -0.2872 | Yes |
| 271 | CDK1 |  |  | 3149 | -0.727 | -0.2835 | Yes |
| 272 | HNRNPD |  |  | 3157 | -0.738 | -0.2821 | Yes |
| 273 | MKI67 |  |  | 3158 | -0.738 | -0.2784 | Yes |
| 274 | ORC6 |  |  | 3159 | -0.738 | -0.2747 | Yes |
| 275 | PRIM1 |  |  | 3160 | -0.739 | -0.2710 | Yes |
| 276 | TK1 |  |  | 3162 | -0.745 | -0.2676 | Yes |
| 277 | RNASEH2B |  |  | 3166 | -0.747 | -0.2649 | Yes |
| 278 | DNAJC9 |  |  | 3167 | -0.747 | -0.2611 | Yes |
| 279 | MCM8 |  |  | 3178 | -0.758 | -0.2605 | Yes |
| 280 | CDT1 |  |  | 3179 | -0.758 | -0.2567 | Yes |
| 281 | MCM3 |  |  | 3182 | -0.761 | -0.2536 | Yes |
| 282 | USP1 |  |  | 3183 | -0.762 | -0.2498 | Yes |
| 283 | BRCA1 |  |  | 3185 | -0.765 | -0.2463 | Yes |
| 284 | CDCA2 |  |  | 3189 | -0.767 | -0.2434 | Yes |
| 285 | PSMC3IP |  |  | 3194 | -0.772 | -0.2408 | Yes |
| 286 | XRCC2 |  |  | 3197 | -0.777 | -0.2376 | Yes |
| 287 | CIP2A |  |  | 3199 | -0.782 | -0.2340 | Yes |
| 288 | PXMP2 |  |  | 3200 | -0.782 | -0.2301 | Yes |
| 289 | BARD1 |  |  | 3201 | -0.783 | -0.2262 | Yes |
| 290 | SGO1 |  |  | 3202 | -0.783 | -0.2223 | Yes |
| 291 | CDCA7 |  |  | 3203 | -0.785 | -0.2184 | Yes |
| 292 | CDC6 |  |  | 3204 | -0.785 | -0.2145 | Yes |
| 293 | BIRC5 |  |  | 3208 | -0.793 | -0.2115 | Yes |
| 294 | FBXO5 |  |  | 3212 | -0.797 | -0.2084 | Yes |
| 295 | DIAPH3 |  |  | 3213 | -0.800 | -0.2045 | Yes |
| 296 | CARHSP1 |  |  | 3214 | -0.801 | -0.2005 | Yes |
| 297 | RRM1 |  |  | 3215 | -0.801 | -0.1965 | Yes |
| 298 | SPC25 |  |  | 3219 | -0.806 | -0.1934 | Yes |
| 299 | UBE2C |  |  | 3225 | -0.812 | -0.1909 | Yes |
| 300 | NUSAP1 |  |  | 3237 | -0.825 | -0.1903 | Yes |
| 301 | FOXM1 |  |  | 3238 | -0.826 | -0.1862 | Yes |
| 302 | RFC2 |  |  | 3245 | -0.839 | -0.1839 | Yes |
| 303 | DTL |  |  | 3249 | -0.848 | -0.1806 | Yes |
| 304 | CCP110 |  |  | 3262 | -0.870 | -0.1801 | Yes |
| 305 | NRGN |  |  | 3264 | -0.872 | -0.1760 | Yes |
| 306 | INAFM1 |  |  | 3266 | -0.876 | -0.1720 | Yes |
| 307 | CENPM |  |  | 3269 | -0.881 | -0.1682 | Yes |
| 308 | CHAF1A |  |  | 3270 | -0.882 | -0.1638 | Yes |
| 309 | CDCA5 |  |  | 3273 | -0.885 | -0.1601 | Yes |
| 310 | CCNE2 |  |  | 3276 | -0.889 | -0.1563 | Yes |
| 311 | FEN1 |  |  | 3278 | -0.890 | -0.1521 | Yes |
| 312 | H2AFX |  |  | 3282 | -0.898 | -0.1486 | Yes |
| 313 | HELLS |  |  | 3284 | -0.901 | -0.1444 | Yes |
| 314 | KLHL23 |  |  | 3287 | -0.907 | -0.1406 | Yes |
| 315 | E2F1 |  |  | 3289 | -0.911 | -0.1363 | Yes |
| 316 | RANBP1 |  |  | 3290 | -0.911 | -0.1318 | Yes |
| 317 | CDKN3 |  |  | 3293 | -0.916 | -0.1279 | Yes |
| 318 | ZWINT |  |  | 3296 | -0.925 | -0.1239 | Yes |
| 319 | HMGA1 |  |  | 3299 | -0.931 | -0.1199 | Yes |
| 320 | PCNA |  |  | 3319 | -0.963 | -0.1211 | Yes |
| 321 | GNG4 |  |  | 3323 | -0.971 | -0.1172 | Yes |
| 322 | UBE2T |  |  | 3330 | -0.979 | -0.1142 | Yes |
| 323 | ASRGL1 |  |  | 3333 | -0.984 | -0.1099 | Yes |
| 324 | EMP2 |  |  | 3338 | -0.998 | -0.1062 | Yes |
| 325 | MYBL2 |  |  | 3340 | -1.006 | -0.1015 | Yes |
| 326 | MCM4 |  |  | 3341 | -1.006 | -0.0965 | Yes |
| 327 | EZH2 |  |  | 3342 | -1.015 | -0.0914 | Yes |
| 328 | DEK |  |  | 3344 | -1.025 | -0.0867 | Yes |
| 329 | RAD51AP1 |  |  | 3345 | -1.030 | -0.0815 | Yes |
| 330 | PBK |  |  | 3354 | -1.046 | -0.0788 | Yes |
| 331 | MCM10 |  |  | 3356 | -1.050 | -0.0739 | Yes |
| 332 | ESCO2 |  |  | 3367 | -1.088 | -0.0717 | Yes |
| 333 | NCAPG |  |  | 3370 | -1.098 | -0.0668 | Yes |
| 334 | MCM7 |  |  | 3384 | -1.147 | -0.0652 | Yes |
| 335 | GINS2 |  |  | 3386 | -1.156 | -0.0598 | Yes |
| 336 | DHFR |  |  | 3387 | -1.159 | -0.0540 | Yes |
| 337 | HMGB2 |  |  | 3390 | -1.184 | -0.0487 | Yes |
| 338 | CLSPN |  |  | 3391 | -1.184 | -0.0428 | Yes |
| 339 | CENPU |  |  | 3398 | -1.230 | -0.0386 | Yes |
| 340 | HIST1H1E |  |  | 3401 | -1.237 | -0.0330 | Yes |
| 341 | FAM111B |  |  | 3402 | -1.238 | -0.0269 | Yes |
| 342 | MCUB |  |  | 3405 | -1.245 | -0.0213 | Yes |
| 343 | PCLAF |  |  | 3409 | -1.265 | -0.0159 | Yes |
| 344 | ATAD2 |  |  | 3415 | -1.278 | -0.0112 | Yes |
| 345 | RRM2 |  |  | 3420 | -1.297 | -0.0059 | Yes |
| 346 | CSRP2 |  |  | 3425 | -1.330 | -0.0006 | Yes |
| 347 | TYMS |  |  | 3436 | -1.391 | 0.0032 | Yes |
| 348 | HIST1H1D |  |  | 3466 | -1.743 | 0.0027 | Yes |
| 349 | HIST1H4C |  |  | 3493 | -2.248 | 0.0057 | Yes |
Table: GSEA details [plain text format]

  

Fig 2: DOROSZKO\_U3180MG\_CLUSTER\_3: Random ES distribution      
 Gene set null distribution of ES for **DOROSZKO\_U3180MG\_CLUSTER\_3**
